# Removal of AO unreamed tibial nail with a threaded rod from the Taylor Spatial Frame

**DOI:** 10.1308/003588412X13373405387096b

**Published:** 2012-05

**Authors:** N Heidari, B Riemer

**Affiliations:** University Hospitals Bristol NHS Foundation Trust,UK

**Figure 1:**
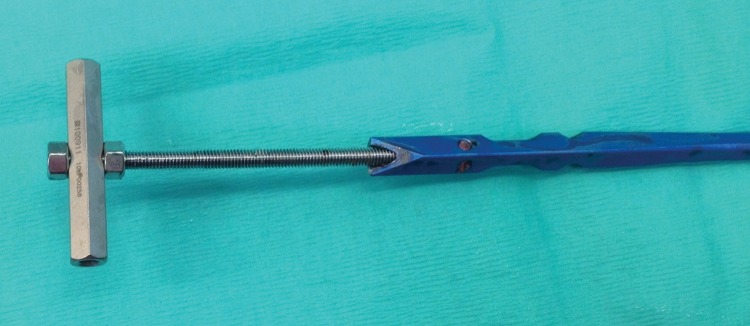
Hexagonal post

Successful removal of metalwork requires a skilled surgeon and the correct instruments. We describe a simple method for the removal of an AO (Arbeitsgemeinschaft für Osteosynthesefragen) unreamed tibial nail in the absence of the correct extraction bolt. The threaded rods from the Taylor Spatial Frame fit into the proximal end of the nail perfectly, allowing for the easy extraction of the nail. The addition of a hexagonal post (Fig 1) allows the construct to be used with a slap hammer if required.

